# HDL and associated factors stratified by sex and menopausal status: results from a community-based survey in Taiwan

**DOI:** 10.18632/oncotarget.24677

**Published:** 2018-03-27

**Authors:** Huan-Cheng Chang, Chuan-Fa Hsieh, Disline Manli Tantoh, Pei-Chieh Ko, Ya-Yu Kung, Mei-Chi Lin, Yi-Ching Liaw, Yung-Po Liaw

**Affiliations:** ^1^ Division of Family Medicine, Department of Community Medicine, Landseed Hospital, Taoyuan, Taiwan; ^2^ Department of Health Care Management, Chang Gung University, Taoyuan, Taiwan; ^3^ Department of Medical Education and Research, Landseed Hospital, Taoyuan, Taiwan; ^4^ Center for General Education, Hsin Sheng College of Medical Care and Management, Taoyuan, Taiwan; ^5^ Department of Public Health and Institute of Public Health, Chung Shan Medical University, Taichung, Taiwan; ^6^ Division of Health Management, Landseed Hospital, Taoyuan, Taiwan; ^7^ Institute of Clinical Medicine, National Yang-Ming University, Taipei, Taiwan; ^8^ Department of Family and Community Medicine, Chung Shan Medical University, Taichung, Taiwan

**Keywords:** HDL, menopause, sex, factors, Taiwan

## Abstract

**Aim:**

To investigate factors, especially modifiable factors associated with high-density lipoprotein (HDL) in Taiwanese based on sex and menopausal status.

**Materials and Methods:**

Participants comprised 2022 men and 2392 women (1267 menopausal and 1125 non-menopausal) aged ≥30 years who resided in Pingzhen district, Taoyuan from 2006-2011. Their data, obtained through questionnaires and measurements were retrieved from the Li-Shin Hospital.

**Results:**

Higher HDL was associated with total cholesterol, underweight, and alcohol drinking in both men and women. It was also associated with education, blood group B, and marital status in men as well as with age in women. Moreover, it was associated with total cholesterol, underweight, and age in both menopausal and non-menopausal women. Furthermore, it was associated with marital status in non-menopausal women and alcohol drinking in menopausal women. Lower HDL was associated with triglycerides, low-density lipoprotein (LDL), overweight, obesity, waist-hip ratio (WHR), uric acid, and smoking in both men and women and with coffee drinking in only women. It was also associated with uric acid, triglycerides, LDL, overweight, obesity, WHR, and body fat in both menopausal and non-menopausal women. Moreover, it was associated with coffee drinking in menopausal women.

**Conclusion:**

Modifiable factors associated with HDL differ according to sex and menopausal status. Sex and menopausal status should be considered when implementing lifestyle changes to raise HDL. For example, both men and women should maintain a normal weight as well as quit smoking.

## INTRODUCTION

HDL cholesterol is an important biomarker of health especially cardiovascular and metabolic health [1]. A vital function of HDL is to enhance the reverse cholesterol transport (RCT) pathway which accounts for its anti-atherosclerotic property [2]. In this pathway, cholesterol is carried to the liver for biliary excretion thereby preventing its accumulation in the arterial wall. HDL’s anti-inflammatory, anti-oxidative, anti-apoptotic, anti-infectious, and anti-thrombotic properties are also well known [1, 3–5]. Maintaining HDL at desired levels is of clinical significance. The beneficial minimal cutoff levels of HDL are ≥40 and ≥50 mg/dL for men and women, respectively [6–8]. A desired HDL level (e.g., >40 mg/dL) is a vital therapeutic target in primary and secondary prevention [9]. It is believed that low levels of HDL need to be raised in many patients even if LDL or non-HDL levels are yet to be reduced to the target levels [10]. For instance, for individuals with low HDL and hypertriglyceridemia, metabolic syndrome, type 2 diabetes mellitus or a high cardiovascular risk (>20%), reaching the HDL therapeutic target is recommended [9]. Because of the health impacts of HDL, it is important to study factors that influence it. A greater fraction of these factors is inherited [11–13]. However, socio-demographic and lifestyle factors including age, sex, menopausal status, hormone replacement therapy, exercise, alcohol drinking, BMI, smoking, and diet are non-genetic [11–16]. Lifestyle factors like exercise, alcohol drinking, smoking, BMI, and diet are modifiable and can raise HDL levels if well managed [15]. Raising HDL levels can in turn help in preventing cardiovascular diseases. There are some controversies regarding HDL and its associated factors especially when menopausal status is considered [16–19]. HDL levels have been shown to be significantly different between men and women [17, 20, 21]. This study was conducted to investigate factors, especially modifiable factors associated with HDL under stratification by sex and menopausal status.

## RESULTS

Tables 1 and 2 show the participants characteristics based on sex and menopausal status, respectively. There was a significant difference (P = 0.0021) between the percentage of men and that of women as far as HDL is concerned (Table 1). However, the percentage of menopausal women was not significantly different from that of the non-menopausal women (Table 2). Tables 3 and 4 show the factors associated with HDL stratified by sex and menopausal status, respectively. Higher HDL was associated with total cholesterol, underweight, and alcohol drinking in both men and women. It was also associated with age 50-69 years, education, blood group B, and marital status (single). However, the association with age was significant only in women while that with education, blood group B, and single status was significant only in men. Lower HDL was associated with uric acid, triglycerides, LDL ≥130 mg/dL, BMI (overweight and obesity), waist-hip ratio, and smoking in both men and women and with coffee drinking only in women (Table 3). Based on menopausal status, higher HDL was associated with age (50-59 years), total cholesterol and BMI (underweight) in both menopausal and non-menopausal women (Table 4). In addition, it was associated with marital status (divorced and widowed) and alcohol drinking. However, the association with marital status was only in non-menopausal women while that with alcohol drinking was only in menopausal women (Table 4). Lower HDL was associated with uric acid, triglycerides, LDL ≥130 mg/dL, BMI (overweight and obese), waist-hip ratio, and body fat in both menopausal and non-menopausal women. Moreover, it was associated with coffee drinking. However, this association was only in menopausal women (Table 4).

**Table 1:** Characteristics of male (n = 2,022) and female (n=2,392) participants

Variable	Men n(%)	Women n(%)	P-value
**HDL (mg/dL)**			0.0021
Male ≥40; female ≥50	1,769(87.49)	2,015(84.24)	
Male <40; female <50	253(12.51)	377(15.76)	
**Total cholesterol (mg/dL)**			0.0001
<200	1,042(51.53)	1,095(45.78)	
≥200	980(48.47)	1,297(54.22)	
**Triglycerides (mg/dL)**			<.0001
<150	1,361(67.31)	1,906(79.68)	
≥150	661(32.69)	486(20.32)	
**LDL (mg/dL)**			0.3500
<130	1,168(57.76)	1,415(59.16)	
≥130	854(42.24)	977(40.84)	
**BMI (kg/m**^2^**)**			<.0001
BMI<18.5 (Underweight)	45(2.23)	84(3.51)	
18.5≤BMI<24 (Normal)	780(38.58)	1,226(51.25)	
24≤BMI<27 (Overweight)	707(34.97)	607(25.38)	
BMI≥27 (Obese)	490(24.23)	475(19.86)	
**Waist circumference (cm)**			0.0252
Male <90; female <80	1,341(66.32)	1,509(63.09)	
Male ≥90; female ≥80	681(33.68)	883(36.91)	
**Waist-hip ratio**			<.0001
Male <0.9; female <0.8	1,118(55.29)	1,003(41.93)	
Male ≥0.9; female ≥0.8	904(44.71)	1,389(58.07)	
**Body fat (%)**			<.0001
Male <25; female <30	1,213(59.99)	936(39.13)	
Male ≥25; female ≥30	809(40.01)	1,456(60.87)	
**Age (years)**			<.0001
30-49	811(40.11)	1,099(45.94)	
50-69	846(41.84)	1,197(50.04)	
≥70	365(18.05)	96(4.01)	
**Education**			<.0001
University and above	619(30.61)	410(17.14)	
Senior high	672(33.23)	754(31.52)	
Junior high	320(15.83)	439(18.35)	
Elementary and below	411(20.33)	789(32.98)	
**Blood type**			0.3082
A	531(26.26)	637(26.63)	
B	466(23.05)	519(21.70)	
O	901(44.56)	1,112(46.49)	
AB	124(6.13)	124(5.18)	
**Marital status**			<.0001
Single	126(6.23)	72(3.01)	
Married	1,826(90.31)	2,167(90.59)	
Co-habiting	5(0.25)	8(0.33)	
Divorced	42(2.08)	65(2.72)	
Widowed	22(1.09)	79(3.30)	
Others	1(0.05)	1(0.04)	
**Fasting blood glucose (mg/dL)**			0.2510
<126	1,884(93.18)	2,249(94.02)	
≥126	138(6.82)	143(5.98)	
**Creatinine (mg/dL)**			<.0001
<1.4	1,844(91.20)	2,365(98.87)	
≥1.4	178(8.80)	27(1.13)	
**Uric acid (mg/dL)**			<.0001
Male ≤7; female ≤6	1,351(66.82)	1,948(81.44)	
Male >7; female >6	671(33.18)	444(18.56)	
**AST (U/L)**			<.0001
<40	1,848(91.39)	2,260(94.48)	
≥40	174(8.61)	132(5.52)	
**ALT (U/L)**			<.0001
<40	1,623(80.27)	2,147(89.76)	
≥40	399(19.73)	245(10.24)	
**SBP (mmHg)**			<.0001
<120	630(31.16)	1,222(51.09)	
120-139	856(42.33)	749(31.31)	
≥140	536(26.51)	421(17.60)	
**DBP (mmHg)**			<.0001
<80	1,114(55.09)	1,687(70.53)	
80-89	562(27.79)	468(19.57)	
≥90	346(17.11)	237(9.91)	
**Smoking**			<.0001
Never	1,010(49.95)	2,299(96.11)	
Quit	289(14.29)	22(0.92)	
Current	723(35.76)	71(2.97)	
**Alcohol drinking**			<.0001
Never	1,464(72.40)	2,299(96.11)	
Quit	93(4.60)	8(0.33)	
Current	465(23.00)	85(3.55)	
**Betel nut chewing**			<.0001
Never	1,742(86.15)	2,378(99.41)	
Quit	138(6.82)	5(0.21)	
Current	142(7.02)	9(0.38)	
**Exercise**			0.4399
No	717(35.46)	875(36.58)	
Yes	1,305(64.54)	1,517(63.42)	
**Vegetarian**			<.0001
No	1,874(92.68)	2,132(89.13)	
Yes	148(7.32)	260(10.87)	
**Coffee drinking**			0.0075
<3 times per week	370(18.30)	515(21.53)	
≥3 times per week	1,652(81.70)	1,877(78.47)	
**Disease history**			
Diabetes	146(7.22)	130(5.43)	0.0146
Hypertension	409(20.23)	355(14.84)	<.0001
Heart disease	123(6.08)	92(3.85)	0.0006
Hyperlipidemia	147(7.27)	119(4.97)	0.0014
Stroke	31(1.53)	3(0.13)	<.0001
**Menopause**			
No		1,125(47.03)	
Yes		1,267(52.97)	
**Hormone therapy**			
No		2,145(89.67)	
Yes		247(10.33)	
**Oral contraceptives**			
No		2,281(95.36)	
Yes		111(4.64)	

**Table 2:** Characteristics of non-menopausal (n=1,125) and menopausal (n=1,267) women

Variable	No menopause n(%)	Menopause n(%)	P-value
**HDL (mg/dL)**			0.8829
≥50	949(84.36)	1066(84.14)	
<50	176(15.64)	201(15.86)	
**Total cholesterol (mg/dL)**			<.0001
<200	662(58.84)	433(34.18)	
≥200	463(41.16)	834(65.82)	
**Triglycerides (mg/dL)**			<.0001
<150	979(87.02)	927(73.16)	
≥150	146(12.98)	340(26.84)	
**LDL (mg/dL)**			<.0001
<130	810(72.00)	605(47.75)	
≥130	315(28.00)	662(52.25)	
**BMI (kg/m**^2^**)**			<.0001
BMI<18.5 (Underweight)	56(4.98)	28(2.21)	
18.5≤BMI<24 (Normal)	672(59.73)	554(43.73)	
24≤BMI<27 (Overweight)	237(21.07)	370(29.20)	
BMI≥27 (Obese)	160(14.22)	315(24.86)	
**Waist circumference (cm)**			<.0001
<80	821(72.98)	688(54.30)	
≥80	304(27.02)	579(45.70)	
**Waist-hip ratio**			<.0001
<0.8	604(53.69)	399(31.49)	
≥0.8	521(46.31)	868(68.51)	
**Body fat (%)**			<.0001
<30	536(47.64)	400(31.57)	
≥30	589(52.36)	867(68.43)	
**Age (years)**			<.0001
30-49	966(85.87)	133(10.50)	
50-69	159(14.13)	1,038(81.93)	
≥70	0(0.00)	96(7.58)	
**Education**			<.0001
University and above	327(29.07)	83(6.55)	
Senior high	512(45.51)	242(19.10)	
Junior high	197(17.51)	242(19.10)	
Elementary and below	89(7.91)	700(55.25)	
**Blood type**			0.4452
A	306(27.20)	331(26.12)	
B	254(22.58)	265(20.92)	
O	513(45.60)	599(47.28)	
AB	52(4.62)	72(5.68)	
**Marital status**			<.0001
Single	57(5.07)	15(1.18)	
Married	1,018(90.49)	1,149(90.69)	
Co-habiting	1(0.09)	7(0.55)	
Divorced	39(3.47)	26(2.05)	
Widowed	10(0.89)	69(5.45)	
Others	0(0.00)	1(0.08)	
**Fasting blood glucose (mg/dL)**			<.0001
<126	1,094(97.24)	1,155(91.16)	
≥126	31(2.76)	112(8.84)	
**Creatinine (mg/dL)**			0.0007
<1.4	1,121(99.64)	1,244(98.18)	
≥1.4	4(0.36)	23(1.82)	
**Uric acid (mg/dL)**			<.0001
≤6	1,001(88.98)	947(74.74)	
>6	124(11.02)	320(25.26)	
**AST (U/L)**			<.0001
<40	1,086(96.53)	1,174(92.66)	
≥40	39(3.47)	93(7.34)	
**ALT (U/L)**			<.0001
<40	1,042(92.62)	1,105(87.21)	
≥40	83(7.38)	162(12.79)	
**SBP (mmHg)**			<.0001
<120	733(65.16)	489(38.60)	
120-139	300(26.67)	449(35.44)	
≥140	92(8.18)	329(25.97)	
**DBP (mmHg)**			<.0001
<80	863(76.71)	824(65.04)	
80-89	181(16.09)	287(22.65)	
≥90	81(7.20)	156(12.31)	
**Smoking**			0.0005
Never	1,065(94.67)	1,234(97.40)	
Quit	18(1.60)	4(0.32)	
Current	42(3.73)	29(2.29)	
**Alcohol drinking**			0.1191
Never	1,073(95.38)	1,226(96.76)	
Quit	3(0.27)	5(0.39)	
Current	49(4.36)	36(2.84)	
**Betel nut chewing**			0.2393
Never	1,118(99.38)	1,260(99.45)	
Quit	4(0.36)	1(0.08)	
Current	3(0.27)	6(0.47)	
**Exercise**			<.0001
No	464(41.24)	411(32.44)	
Yes	661(58.76)	856(67.56)	
**Vegetarian**			0.0804
No	1,016(90.31)	1,116(88.08)	
Yes	109(9.69)	151(11.92)	
**Coffee drinking**			<.0001
<3 times per week	321(28.53)	194(15.31)	
≥3 times per week	804(71.47)	1,073(84.69)	
**Disease history**			
Diabetes	21(1.87)	109(8.60)	<.0001
Hypertension	65(5.78)	290(22.89)	<.0001
Heart disease	25(2.22)	67(5.29)	<.0001
Hyperlipidemia	32(2.84)	87(6.87)	<.0001
Stroke	2(0.18)	1(0.08)	0.4954
**Hormone therapy**			<.0001
No	1,086(96.53)	1,059(83.58)	
Yes	39(3.47)	208(16.42)	
**Oral contraceptives**			0.3107
No	1,078(95.82)	1,203(94.95)	
Yes	47(4.18)	64(5.05)	

**Table 3:** Linear regression analysis showing factors associated with HDL concentration based on sex

	Men		Women	
	*β*	P-value	*β*	P-value
**Total cholesterol (Ref: <200)**	-	-	-	-
≥200	11.599	<.0001	14.641	<.0001
**Triglycerides (Ref: <150)**				
≥150	-9.518	<.0001	-12.307	<.0001
**LDL (Ref: <130)**	-	-	-	-
≥130	-7.642	<.0001	-8.701	<.0001
**BMI (Ref: Normal)**	-	-	-	-
Underweight	9.273	<.0001	5.161	0.0003
Overweight	-2.849	<.0001	-2.583	0.0009
Obese	-4.337	<.0001	-2.925	0.0032
**WHR (Ref: Male <0.9 and female <0.8)**	-	-	-	-
Male ≥0.9 and female ≥0.8	-1.657	0.0092	-3.016	<.0001
**Body fat (Ref: Male <25 and female <30)**				
Male ≥25 and female ≥30	-1.167	0.0743	-2.788	<.0001
**Age (Ref: 30-49)**	-	-	-	-
50-69	0.207	0.7470	2.459	0.0035
≥70	0.872	0.3569	2.775	0.0874
**Education (Ref: University and above)**	-	-	-	-
Senior high	2.025	0.0018	-0.207	0.7919
Junior high	1.788	0.0291	0.131	0.8857
Elementary and below	1.661	0.0444	0.647	0.4920
**Blood type (Ref: A)**	-	-	-	-
B	1.590	0.0270	0.937	0.2041
O	-0.245	0.6930	0.219	0.7236
AB	1.048	0.3518	-0.129	0.9158
**Marital status (Ref: Married)**	-	-	-	-
Single	2.333	0.0291	-0.351	0.8189
Co-habiting	1.742	0.7301	-4.630	0.2946
Divorced	1.359	0.4466	2.803	0.0772
Widowed	-1.891	0.4373	-1.008	0.4888
Others	-12.073	0.2891	-8.667	0.4870
**Creatinine (Ref: <1.4)**	-	-	-	-
≥1.4	-2.505	0.0096	-1.997	0.4247
**Uric acid (Ref: Male ≤7 and female ≤6)**	-	-	-	-
Male >7 and female >6	-2.020	0.0004	-2.784	<.0001
**Smoking (Ref: Never)**	-	-	-	-
Quit	1.578	0.0423	-0.137	0.9605
Current	-1.591	0.0087	-3.247	0.0393
**Alcohol drinking (Ref: Never)**	-	-	-	-
Quit	1.560	0.2098	5.099	0.2650
Current	3.695	<.0001	3.355	0.0204
**Vegetarian (Ref: No)**	-	-	-	-
Yes	-1.071	0.2676	-2.038	0.0136
**Coffee drinking (Ref: ≥3 times per week)**	-	-	-	-
<3 times per week	0.931	0.1627	-1.823	0.0043

Adjusted for fasting blood glucose, GOT, GPT, SBP, DBP, betel nut chewing, exercise, diabetes, hypertension, heart disease, hyperlipidemia, stroke, menopausal status, hormone therapy, and oral contraceptives.

**Table 4:** Linear regression analysis showing factors associated with HDL concentration based on menopausal status

	No menopause		Menopause	
	*β*	P-value	*β*	P-value
**Total cholesterol (Ref: <200)**	-	-	-	-
≥200	15.759	<.0001	13.379	<.0001
**Triglycerides (Ref: <150)**	-	-	-	-
≥150	-11.907	<.0001	-12.268	<.0001
**LDL (Ref: <130)**	-	-	-	-
≥130	-9.975	<.0001	-7.494	<.0001
**BMI (Ref: Normal)**	-	-	-	-
Underweight	5.274	0.0032	4.569	0.0627
Overweight	-3.386	0.0041	-2.024	0.0553
Obese	-5.383	0.0006	-1.546	0.234
**WHR (Ref: <0.8)**	-	-	-	-
≥0.8	-2.273	0.0144	-3.697	<.0001
**Body fat (Ref: <30)**	-	-	-	-
≥30	-3.169	0.0016	-2.347	0.0202
**Age (Ref: 30-49)**	-	-	-	-
50-69	2.461	0.0404	2.522	0.0388
≥70	-	-	3.385	0.0680
**Education (Ref: University and above)**	-	-	-	-
Senior high	-0.145	0.8741	-0.219	0.8917
Junior high	1.742	0.1461	-1.906	0.2343
Elementary and below	-0.880	0.5909	0.274	0.8561
**Blood type (Ref: A)**	-	-	-	-
B	1.334	0.2097	0.794	0.4432
O	1.575	0.0825	-1.127	0.1898
AB	1.017	0.5870	-1.120	0.4892
**Marital status (Ref: Married)**	-	-	-	-
Single	0.328	0.8514	-1.937	0.5540
Co-habiting	12.639	0.3111	-7.118	0.1331
Divorced	4.194	0.0462	0.296	0.9061
Widowed	9.034	0.0262	-2.491	0.1137
Others	-	-	-9.490	0.4486
**Creatinine (Ref: <1.4)**	-	-	-	-
≥1.4	5.574	0.4177	-2.444	0.3681
**Uric acid (Ref: ≤6)**	-	-	-	-
>6	-3.525	0.0049	-2.620	0.0031
**Smoking (Ref: Never)**	-	-	-	-
Quit	0.466	0.8815	-4.770	0.4599
Current	-3.605	0.0759	-2.491	0.3288
**Alcohol drinking (Ref: Never)**	-	-	-	-
Quit	8.360	0.2864	1.784	0.7611
Current	2.909	0.1267	4.565	0.0436
**Vegetarian (Ref: No)**	-	-	-	-
Yes	-2.320	0.0674	-2.024	0.0659
**Coffee drinking (Ref: ≥3 times per week)**	-	-	-	-
<3 times per week	-0.969	0.2499	-2.987	0.0028

Adjusted for fasting blood glucose, GOT, GPT, SBP, DBP, betel nut chewing, exercise, diabetes, hypertension, heart disease, hyperlipidemia, stroke, hormone therapy, and oral contraceptives.

## DISCUSSION

In the current study, factors associated with HDL were determined based on sex and menopausal status. Based on sex, there were significant differences between men and women as far as HDL is concerned. To our knowledge, this study is among the first in Taiwan to stratify the factors that are associated with HDL by sex and menopausal status. A study investigated the effects of gender and menopausal status on plasma lipoprotein subspecies and particle sizes [22]. However, its aim differs from that of the current study in terms of the outcome since this study’s outcome was only HDL. Moreover, some previous studies conducted in women focused generally on cardiovascular risk factors and lipid profiles not specifically HDL [23–25]. It is well known that HDL levels in men and women significantly differ [17, 20, 21, 26]. One reason for this is the influence of estrogen and testosterone on the activities of hepatic lipase. Hepatic lipase plays a role in HDL metabolism and its levels are inversely related with those of HDL [27]. Estrogen and testosterone respectively tend to decrease and increase hepatic lipase levels [26, 28]. As a result, women tend to have higher HDL levels than men [17, 20, 21, 26]. In this study, the percentage of menopausal women was not significantly different from that of the non-menopausal women. Several studies on the association between HDL and menopausal status have shown controversial results [16-19, 29]. It is still unclear whether the relationship between HDL and menopausal status is due to hormone levels [30, 31]. However, estrogen is believed to be associated with higher HDL levels [28, 32]. At post menopause, androgen levels are higher than estrogen levels thereby leading to relatively lower levels of HDL [31, 32]. HDL, body fat, waist circumference, BMI, triglyceride, and LDL are known risk factors of metabolic syndrome [33, 34]. Triglyceride, LDL, higher BMI, increased waist circumference, and body fat have been associated with lower HDL while lower BMI and total cholesterol have been associated with higher HDL [11, 20, 26, 35–37]. Among the metabolic biomarkers mentioned above, total cholesterol, triglyceride, LDL, and waist-hip ratio were associated with HDL in both sexes and menopausal statuses in this study. However, BMI and body fat did not yield similar results based on our stratification. For instance, the different BMI categories were associated with HDL in both men and women. That is, underweight was significantly associated with higher HDL while overweight and obesity were significantly associated with lower HDL in both men and women. Stratification by menopausal status yielded similar results. However, significant results were prominent only in non-menopausal women. It is unclear why significant associations were found only in non-menopausal women. Body fat was associated with lower HDL only in women. However, it was associated with lower HDL in both menopausal and non-menopausal women. Again, it is still unclear why there was no significant association in men. So far, the associations between age and HDL have not been consistent. For instance, in some studies, higher HDL levels were observed among older African Americans especially females [38] and Chinese adults [39]. However, in another study, a significant association between age and higher HDL was prominent only among Hong Kong Chinese females [40]. In this study, the association between HDL and age was stronger in women (higher *β*) than men in all age groups. However, a significant association was found only in women aged 50-69 years. A study showed higher levels of HDL in those aged 60-69 years [21]. The prominent association between age and HDL only in women might be due to estrogen. However, at age 50-69 years, estrogen levels are believed to be relatively lower than those at younger ages. Therefore, this association might not be explained in terms of estrogen levels. This greater influence might have been due to higher baseline HDL in females. In a study, educational level was associated with lower HDL in men, though not in a significant manner [26]. However, in another study, it was significantly associated with higher HDL [41]. In the current study, all educational levels were associated with higher HDL levels in the male participants. The impact of education on HDL seems to be in an indirect manner. It is thought that education affects the modifiable factors like smoking, drinking, and exercise which in turn affect HDL levels [26, 41]. Based on this knowledge, the existence of a significant association only in men can be explained. For instance, there was a positive association between HDL and quitting smoking in men only (Table 3). Moreover, the association between alcohol drinking and HDL was stronger in men than women (Table 3). Non-O blood groups have been associated with an increased risk of cardiovascular diseases [42, 43]. On the other hand, all blood groups have been associated with low levels of HDL and therefore, higher cardiovascular risk profiles [44]. In contrast, only blood group B was associated with higher HDL in this study specifically among men. It is not certain if this might have been due to some genetic factors. Therefore, future studies should elaborately investigate this. In this study, higher HDL was associated with marital status (for single men as well as divorced and widowed non-menopausal women). In a previous study, HDL was higher in single than married men and women but statistical significance was achieved only in men [45]. In another study, there was an association between HDL and marital status [11]. Similar to our results, statistical significance was prominent only among single men. Furthermore, in another study, the HDL of married women was not significantly different from that of the divorced or widowed women [46]. It is not very clear why a significant association was prominent only in single men in the current study. However, this might partly be due to some modifiable factors like BMI which can be managed by physical activity. Single men are more likely to be more physically active than single women. This can help in lowering the BMI as well as increasing HDL levels. Furthermore, it is not very clear why significant associations were prominent only in divorced and widowed non-menopausal women in the current study. One of the reasons could be that non-menopausal females are likely to have higher HDL levels at baseline when compared to menopausal females due to differences in estrogen levels. Moreover, most of the menopausal females are younger and can also engage in more exercise relative to the menopausal women. Similar to the current study, creatinine and uric acid have been significantly associated with lower HDL (29-31). As previously shown, cigarette smoking is a well-known risk factor for low HDL [7, 11, 14, 16, 40]. However, moderate alcohol consumption is associated with higher HDL [8, 11, 15, 20, 37, 40, 47]. Similarly, alcohol consumption was associated with higher HDL among both sexes. Based on menopausal status, a significant association was observed only in non-menopausal females. The reason for the absence of a prominent association in menopausal females cannot be explicitly stated. Despite the positive association of alcohol with HDL, only moderate amounts should be consumed due to other health issues associated with alcohol drinking. Like our results, a meta-analysis of observational studies and clinical trials showed that a plant-based vegetarian diet reduced HDL cholesterol [48]. However, another meta-analysis showed no significant association between HDL and vegetarian diet [49]. Coffee drinking was shown to enhance the HDL mediated reverse cholesterol transport [50]. One of the reasons for cholesterol efflux from macrophages is the presence of phenolic acids in coffee [50]. However, coffee also contains kahweol and cafestol which are believed to increase cholesterol. In this study, coffee consumption was associated with decreased HDL in only women and this has previously been shown [37]. Stratification by menopausal status yielded similar results. However, this was only in non-menopausal women. The reason why the association between HDL and coffee was significant only in women, especially non-menopausal women is unknown. Our study was limited in its cross-sectional nature.

## MATERIALS AND METHODS

This study included 4414 participants comprising 2022 men and 2392 women who were 30 years and above. Of the female participants, 1267 were menopausal while 1125 were non-menopausal. The participants resided in the Pingzhen district of Taoyuan city from 2006 to 2011. Their data were retrieved from the Li-Shin Hospital, a regional hospital in Northern Taiwan. These data included age, education, blood type, marital status, fasting blood glucose, creatinine, uric acid, total cholesterol, triglycerides, HDL, LDL, aspartate transaminase (AST), alanine transaminase (ALT), systolic blood pressure (SBP), diastolic blood pressure (DBP), BMI, waist circumference, waist-hip ratio, body fat, smoking, alcohol drinking, betel nut chewing, exercise, vegetarian diet, coffee drinking, disease history, menopausal status, hormone therapy, and oral contraceptives. Chi-square test was used to compare the percentage of men with women as well that of menopausal with non-menopausal women. Multiple linear regression analysis was used to determine the relationship between HDL and the other variables. All statistical analyses were performed with SAS 9.4 (SAS Institute, Cary, NC, USA). This study was approved by the Antai Medical Care Cooperation Antai Tian-Sheng Memorial Hospital Institutional Review Board (No. 16-006-C0).

## CONCLUSIONS

Modifiable factors associated with HDL differ according to sex and menopausal status. For example, LDL, triglycerides, and WHR were associated with HDL in both sexes and menopausal statuses. BMI, drinking, and smoking were associated with HDL in both sexes but not in both menopausal statuses. Vegetarian diet and coffee drinking were associated with HDL only in women but not in both menopausal statuses. Sex and menopausal status should be considered when implementing lifestyle changes to raise HDL. For example, to achieve desirable levels of HDL, it is vital for both men and women to maintain a normal weight as well as quit smoking.
